# Previously undiagnosed scoliosis presenting as pleuritic chest pain in the emergency department – a case series and a validating retrospective audit

**DOI:** 10.1186/s12873-021-00455-x

**Published:** 2021-05-17

**Authors:** Gabor Xantus, Derek Burke, Peter Kanizsai

**Affiliations:** 1 Clinical Centre, University of Pécs, Pecs, Hungary; 2 Gibraltar University, Gibraltar, GX11 1AA Gibraltar

**Keywords:** Chest pain, Emergency medicine, Musculoskeletal, Safe discharge, costo-chondritis

## Abstract

**Background:**

Chest pain is one of the commonest presenting complaints in urgent/emergency care, with a lifelong prevalence of up to 25% in the adult population. Pleuritic chest pain is a subset of high investigation burden because of a diverse range of possible causes varying from simple musculoskeletal conditions to pulmonary embolism.

**Case series:**

Among otherwise fit and healthy adult patients presenting in our emergency department with sudden onset of unilateral pleuritic chest pain, within 1 month we identified a cohort of five patients with pin-point tenderness in one specific costo-sternal joint often with referred pain to the back. All cases had apparent and, previously undiagnosed mild/moderate scoliosis.

**Methods:**

To confirm and validate the observed association between scoliosis and pleuritic chest pain, a retrospective audit was designed and performed using the hospital’s electronic medical record system to reassess all consecutive adult chest pain patients.

**Results:**

The Odds Ratio for having chest pain with scoliosis was 30.8 [95%CI 1.71–553.37], twenty times higher than suggested by prevalence data.

**Discussion:**

In scoliosis the pathologic lateral curvature of the spine adversely affects the functional anatomy of both the spine and ribcage. In our hypothesis the chest wall asymmetry enables minor slip/subluxation of a rib either in the costo-sternal and/or costovertebral junction exerting direct pressure on the intercostal nerve causing pleuritic pain.

**Conclusion:**

Thorough physical examination of the anterior and posterior chest wall is key to identify underlying scoliosis in otherwise fit patients presenting with sudden onset of pleuritic pain. Incorporating assessment for scoliosis in the low-risk chest pain protocols/tools may help reducing the length of stay in the emergency department and, facilitate speedy but safe discharge with increased patient satisfaction.

## Background

Chest pain is one of the commonest presenting complaints both in primary and urgent/emergency care, with a lifelong prevalence of up to 25% [1] with a very high investigation burden and consumes valuable resources [2]. Sudden onset of pleuritic chest pain may herald pulmonary embolism (PE), a condition which is not infrequently left undiagnosed in the acute care setting [3]. This is a condition of significant mortality (8–30%) if not recognised early and treated appropriately [4]. Despite the use of diagnostic tools like the Pulmonary Embolism Rule-out Criteria (PERC) [5] and the revised Geneva [6] or Wells’ score [7], risk-stratification and diagnosis are often challenging due to a large number of mimicks; especially in those aged 50 years and over and women taking exogenous oestrogen supplements [8].

Patients presenting with pleuritic chest pain with marginally abnormal scores in the emergency department (ED) are often subjected to 12 lead ECG, bloodwork and, if they fall into higher probability/clinical suspicion, may undergo either contrast mediated multi-slice computed tomography or thrombo-prophylaxis [9]. Even with no riks/low risk for PE, these patients often discharged after having a “safety net” D-dimer and/or Troponin assay especially if the attending physicians do not comply with evidence-based rational diagnostic strategies and/or the department is crowded [10, 11].

Chest wall tenderness is not unusual in PE because of reactive pleurisy. Physicians therefore have repeatedly been cautioned not to dismiss the possibility of a PE because of reproducible, seemingly musculoskeletal chest pain [12].

Among patients presenting with sudden onset of pleuritic chest pain, we seemed to have observed a low risk subset with distinct commonalities; all reported sudden, non-traumatic, unilateral localised pain with or without subjective shortness of breath.

### Objectives

To determine the cause of in this cohort we decided that all consecutive low risk chest pain patients in the month of November 2020 would be assessed identically using a prospectively predefined structured history taking template and would be subjected to thorough physical examination. We hoped that establishing a series of homogenous cases might help us generating a viable clinical hypothesis. We also decided that if such a cohort can be set up, all similar cases of the past 6 months would be audited retrospectively to validate our cross-sectional findings.

## Case series

### Presentation

**Fig. 1:**
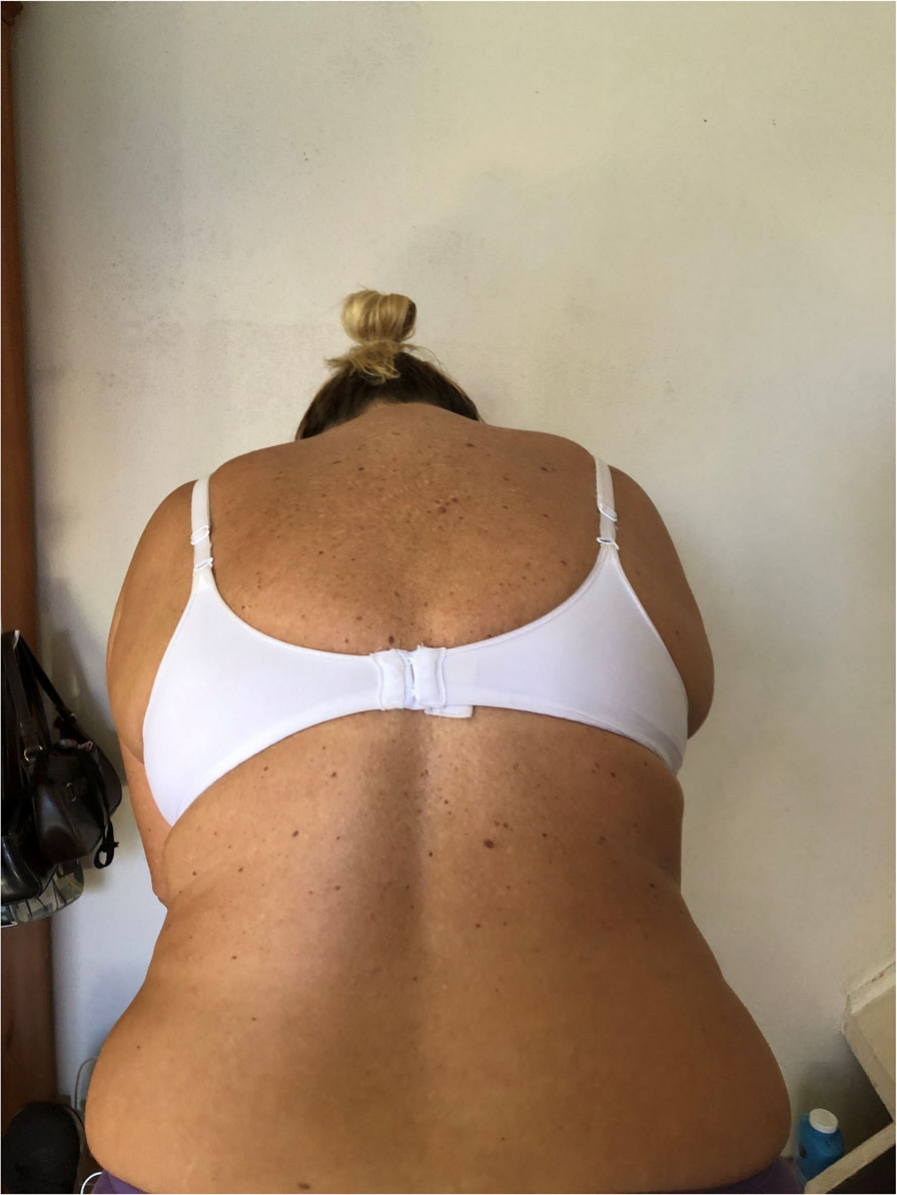
Caption: Dorsal tilt secondary to a rib hump when leaning forward (Adams test) in scoliosis

**Fig. 2:**
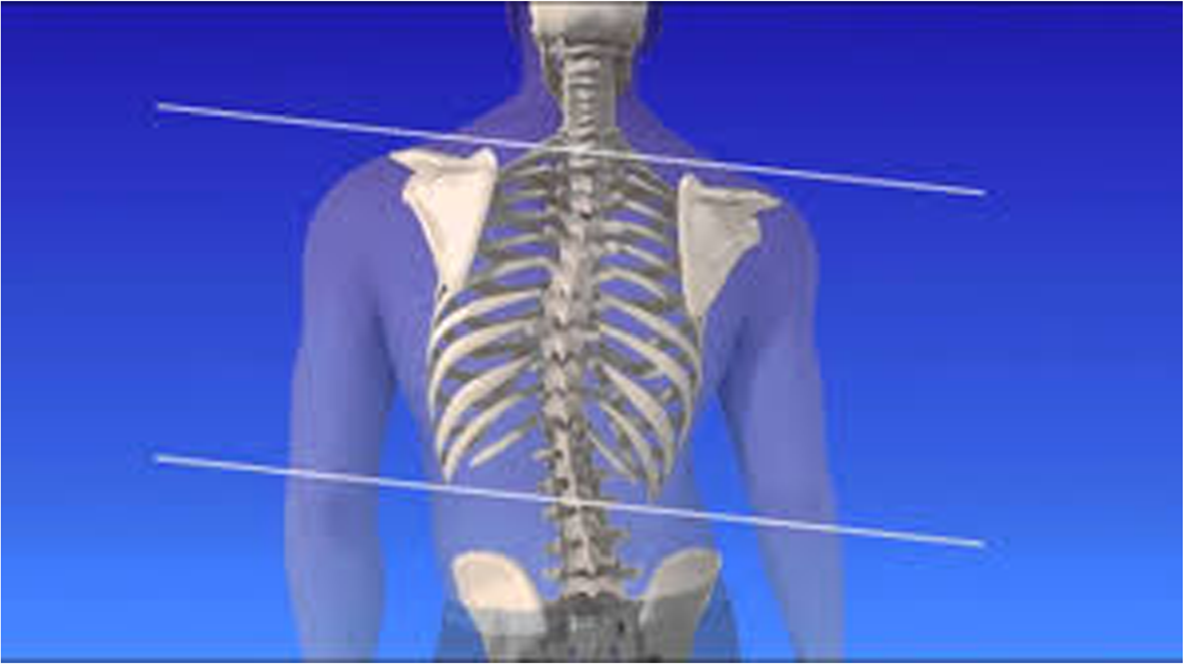
Caption: Difference in horizontal distance between the shoulder blades and the vertebral column in scoliosis

**Fig. 3:**
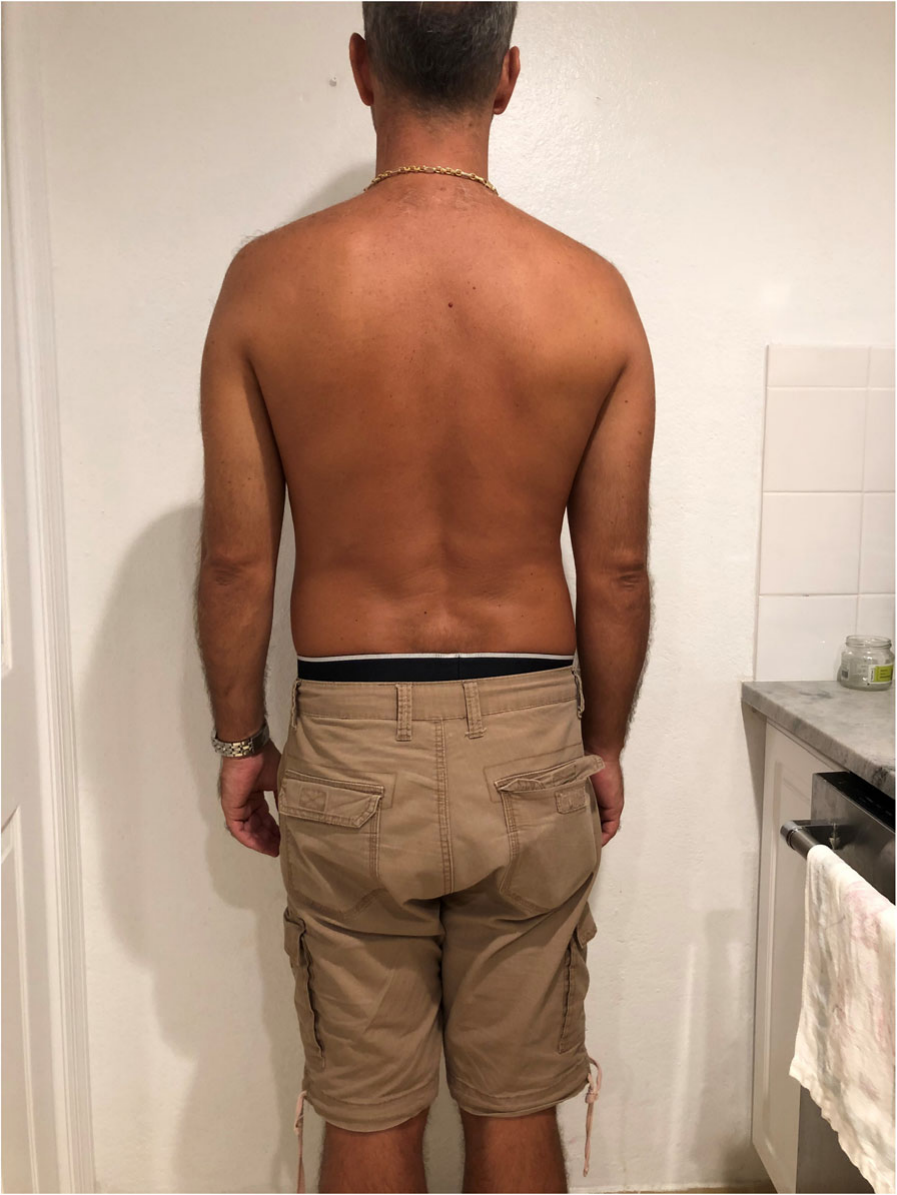
Caption: Difference in vertical distance between the shoulder blades and the posterior iliac spine in scoliosis

**Fig. 4:**
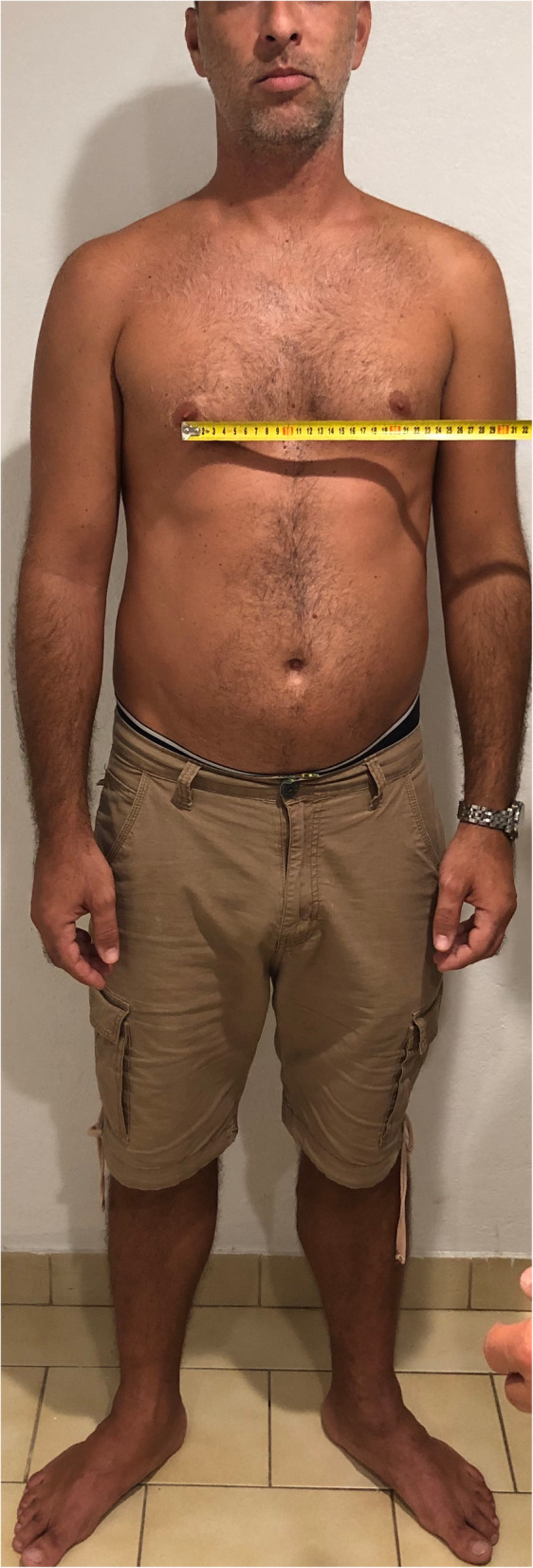
Caption: Asymmetrical sterno-clavicular angle in scoliosis

During the above period in our small district emergency department (ED) with the yearly census of 30,000/year we recorded a case series of five adults and one nine-year old child presenting with low-risk chest pain. Low-risk was established at triage based on the Manchester Triage and TIMI score [13, 14]. The patients were seen by 3 independent clinicians (GX provided second opinion in 2 cases). All patients reported pain on palpation either in the 4th–5th costo-sternal and/or costovertebral junction of the same segment, although less so in the latter. Interestingly, in this cohort we found clinically obvious scoliosis in each patient with apparent asymmetry of the thoracic spine and the rib cage. The scoliosis was mild/moderate in all patients however, with distinctive features well documented in the orthopaedic literature [15]. All patients reported sudden onset of unilateral pain confined to side of the chest only either the 4th or the 5th costo-sternal junction sometimes with referred pain in the ipsilateral costo-vertbral junction of the same segment. In one patient only the latter was present. The pain had pin-point localisation, while all other parts of the chest wall were pain- free, including the contralateral costo-sternal junction. We observed the following typical changes in the thoracic spine, the shoulder girdle and the rib cage anatomy associated with scoliosis: All demonstrated (variable degree) dorsal tilt secondary to a rib hump when leaning forward (Adams test) [Fig. 1], with notable asymmetry of the shoulder blades as well. There were obvious differences in distance both horizontally between the shoulder blades and the vertebral column [Fig. 2]**,** and vertically between the tip of the scapula and the spina iliaca posterior [Fig. 3]. We observed apparent asymmetry of the rib cage, with the nipple line deviating from the horizontal plane and the asymmetrical sterno-clavicular angle [Fig. 4]. All patients were discharged with the diagnosis of costo-chondritis, which is the closest diagnostic label in the drop-down discharge menu to musculoskeletal (MSK) chest pain in our electronic medical record (EMR). None of them presented later with shingles, so herpetic origin was excluded.

## Results

All adult patients (5/5) reported sudden onset of very intense (stabbing or knife like) pain, which was localised only on one side of the chest. Four out of five complained of difficulty in breathing (“catching my breath”, “stitching”). Upon further questioning the breathing difficulty was reported to be due to pain with patients struggled to take deep breaths and therefore, subjectively feeling short of breath. All had normal pulse oximetry values (> 94%) clear chests and equal breath sounds. If the onset of pain was a day prior to the index visit, patients often mentioned difficulty in sleeping as they could not find a comfortable position in bed due to back or chest pain when lying flat (3/5). Two patients reported previous similar episodes of chest pains, one even underwent investigation for pulmonary embolism. Almost all patients used NSAIDs (4/5) with little or no effect and all denied prior knowledge of their spinal condition (5/5). Those investigated previously said that they had not been asked to remove clothing from the upper body during examination.

## Methods

Even though we have documented our cases in line with the recommendation of the Joanna Briggs Institute [16], still case series is regarded low level of evidence however, all agrees that it is invaluable in generating hypothesis. To correct the obvious limitations of our observations and to validate/confirm the association between the observed clinical variables either a prospective multicentre validation study or an audit of a broader time period might be designed. Due to feasibility, we have chosen the latter and performed a retrospective audit on patients presenting with chest pain and discharged from our department with the diagnosis of costo-chonditis between 01.06.2020 and 12.12.2020. “Chosto-chondritis” was chosen as in our hospital EMR this was the closest match with low-risk chest pain (the drop down discharge menu has no other option for “non-cardiac” or “musculoskeletal” chest pain). The audit covered all consecutive adults (above 18 years of age) seen by senior doctors (staff grade or above).

## Results

In the above 6 months period using the filter option of e-audit function of the EMR (Symphony), we identified 31 patients matching the inclusion criteria. Fifty-two percent were female with the median age 42.5 ± 5. The median length of stay in the ED was 2.01 ± 0.79 h. All had ECG (as per department protocol) and during the index presentation nearly half of them was subjected to blood samples (42% for high-sensitivity troponin and 10% for D-Dimer, 5% both) none of which revealed any significant abnormality. Five patients (16%) had chest X-ray (CXR) during the work up. Interestingly, in seventeen cases (55%) it was documented that the pain was made worse by certain movements and/or taking a deep breath. Seven patients (22.5%) reported to have felt pain on lying on their back or their side. None of the notes made reference to scoliosis or any other obvious deformity of the chest or back. Upon interviewing the doctors who saw the patients involved in the audit all denied having examined the patient’s bare chest and/or back for deformity during their examination. After obtaining a waiver from the hospitals’ Ethics Committee we also cross referenced the available radiographic images of each patient identified by the audit. Twenty-one patients had previous CXR, two had previous thoracic (dorsal) spine plane films and four had no images at all. In ten patients (35.7%) there was obvious radiological evidence of scoliosis. This is nearly a twenty-fold increase in scoliosis prevalence comparing to normal adult population [17]. The Odds Ratio for having chest pain with scoliosis was 30.764 [95%CI 1.71–553.37].

## Discussion

**Fig. 5:**
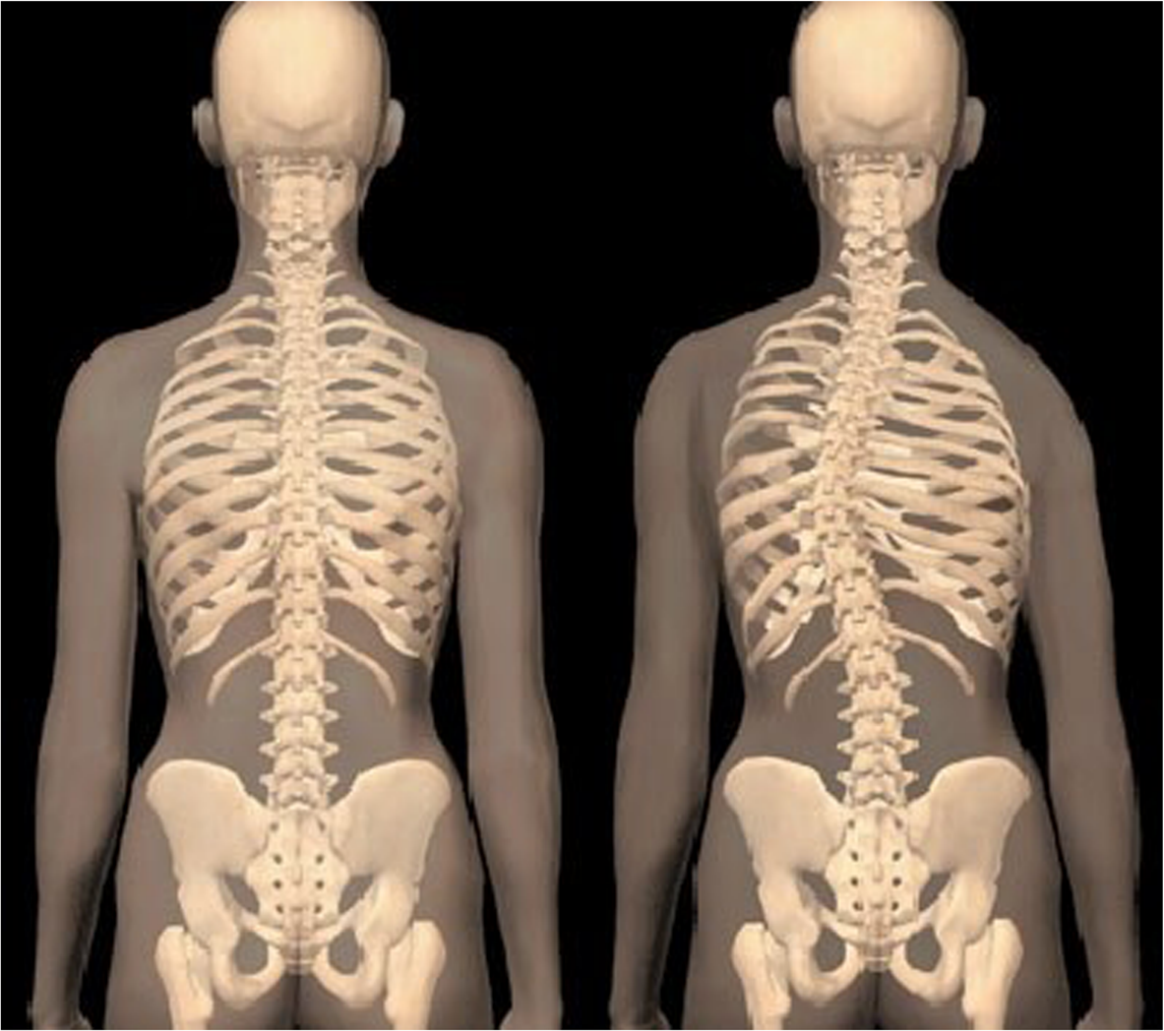
Caption: Significant differences between the angle of the ribs to the vertebra in scoliosis

**Fig. 6:**
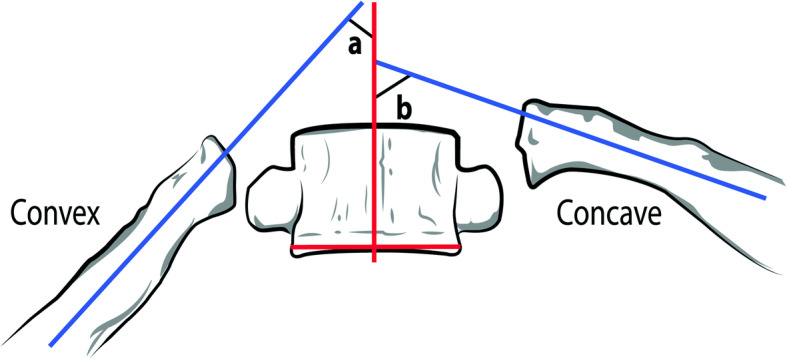
The top of the convexity of curvature is the weakest spot (upper end vertebra)

**Fig. 7:**
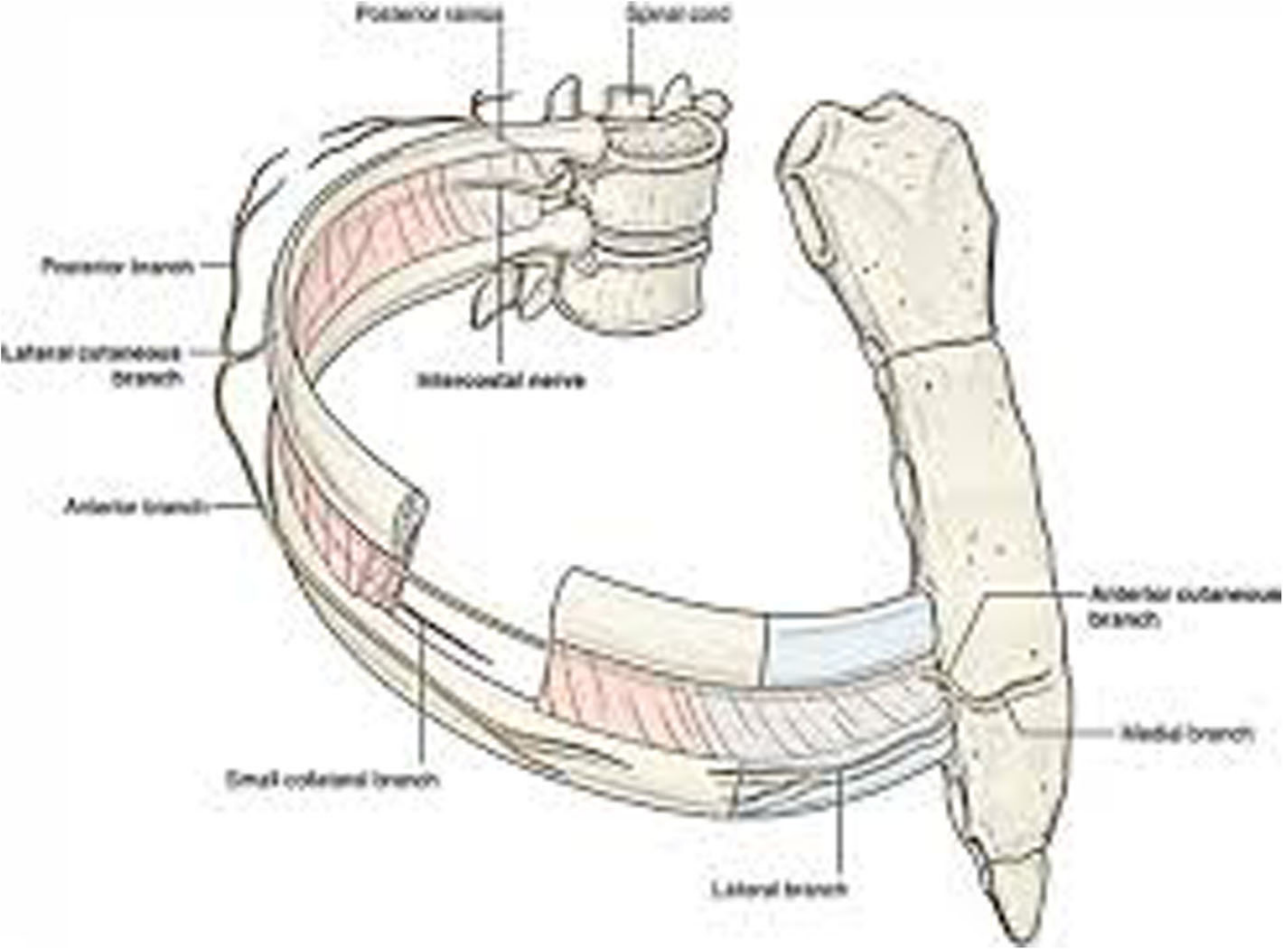
The anatomy of the intercostal structures

To the best of our knowledge, scoliosis in association with MSK chest pain has not been researched in the remits of emergency medicine. Interestingly though, pleurisy is known to cause acute, transient scoliosis [18, 19]. We assume, that in certain cases of sudden onset of pleuritic chest pain/chest wall tenderness, scoliosis might probably play aetiological role due to subluxation of the rib. In most cases of scoliosis, the ribs length is not significantly different between the two sides, [20] unlike the angle of the ribs to the vertebra [Fig. 5]. However, the sideway curvature of the spine causes unequal tensions in the costo-sternal and/or in the ipsilateral corresponding costo-vertebral junctions of the asymmetric rib cage. In consequence, even minor triggers may force one of the rib heads (in our experience typically either the 4th or the 5th) to subluxate from its sternal or vertebral socket in the side of the convexity of curvature at the weakest spot; typically, at the level of the top of the pathologic sideways curvature (upper end vertebra) [Fig. 6]. Even a minuscule displacement in this tight joint would alter the course of the rib and may apply intermittent pressure (mostly during inspiration or Valsalva manoeuvre) to the intercostal nerve running underneath [Fig. 7]. The triggering stimuli are often so mild (like backward stretch of the arm or interlocking the fingers behind the back) that most patients do not attribute the painful episodes to any specific prior movements or injury. On close observation a subtle lump is often visible in the aspect of the pain. We opine that this condition is similar to the “slipping rib syndrome” [21, 22], which usually affects the lower ribs and occurs when the cartilaginous part of a lower rib separates from the bony part causing pain in the chest and sometimes in the upper abdomen. In both cases, the chest pain is probably nociceptive: due to direct, intermittent pressure on the nerve. This mechanism was well explained by previous cadaver models on rib head displacement and costo-sternal mobility [23].

We opine that recognizing the relationship between scoliosis and pleuritic chest pain of probably musculoskeletal origin could create a more effective treatment plan (manipulation, education and/or referral to physiotherapy/chiropractics) in this subgroup increasing patient satisfaction and facilitating speedy, safe discharge. Such holistic treatment might also prevent further unplanned returns with the same presentation as evidence suggests that most unplanned returns related to chest pains are attributed to lack of communication and failure to improve [24]. If our findings were validated by larger, multicentre observations, the presence of scoliosis could potentially be integrated in the work up/management algorithm of low-risk pleuritic chest pains alongside such diagnostic tools like PERC, Wells’ or Geneva score. Safe protocols may help reduce the investigation burden in this subset of chest pain patients and facilitate early discharge with high satisfaction. Additionally, further validation studies may be required to evaluate if the recognition of a broader range of musculoskeletal conditions might make a useful addition to diagnostic tools used in the work up/management of low-risk chest pain (Table 1).

**Table 1:** Clinical tools to aid diagnosis of PE with our recommendation for inclusion

	Geneva score		PERC index		Wells’ score	
**Age**	> 65	+ 1	≥50	+ 1	N/A	
**Previous VTE**	yes	+ 3	yes	+ 1	yes	+ 1.5
**Surgery (under general anaesthesia) or lower limb fracture in past month**	yes	+ 2	yes	+ 1	yes	+ 1.5
**Active malignant condition**	yes	+ 2	N/A		yes	+ 1
**Unilateral lower limb pain**	yes	+ 3		+ 1	yes	+ 3
**Haemoptysis**	yes	+ 2	yes	+ 1	yes	+ 1
**Heart rate**	> 65	+ 3	≥100	+ 1	≥100	+ 1.5
	> 95	+ 5				
**Pain on lower limb palpation and unilateral oedema**			yes	+ 1		
**O**_**2**_ **saturation (room air)**	N/A		< 95%	+ 1	N/A	
**Hormone use**	N/A			+ 1	N/A	
**PE is #1 diagnosis OR equally likely**					yes	+ 3
**Pulmonary embolism is likely**	< 4 points low risk (7–9%) 4–10 points moderate risk (30%) > 11 points high risk (> 60%)		< 1 the likelihood of PE is < 2%. 1 or above PERC cannot rule out PE		< 2 low risk (1.3%) > 2 moderate risk (16.2%) > 6 points high risk (> 40.6%)	
**If the patient is low risk, consider the presence of obvious chest wall/spinal deformity before further investigation**						

Our presumed model may also explain the unsatisfactory pharmacological treatment of patients discharged with the diagnosis of Tietze syndrome, or idiopathic costo-chondritis. We opine that sudden onset of low-risk pleuritic chest pain with apparent scoliosis, is likely to be an acute-on-chronic problem. The management therefore, should be different in the acute stage (when the time elapsed between onset and attendance does not exceed 6 h) than in cases with either longer elapsed time or of high recurrence (subacute or chronic). In acute cases, the rib can be relocated in a fashion similarly to the repositioning of the radial head (nursemaid elbow), a quick manipulation can realign the costal head. We have reviewed sport medicine, physiotherapy and chiropractic literature with keywords of “slipping rib”, “popped rib”, “subluxed rib”. It appears that in the condition is known as subluxed/dislodged rib (“popped out”) and can easily be reversed [25]. The manipulation is simple, and can be done by either a health care practitioner or by the patients themselves. If applied early on, manipulation can completely eliminate the pain. Further studies might be necessary in urgent/emergency care settings to assess feasibility.

### Limitations

Our study has numerous limitations. Case series is inherently biased by convenience sampling, which we have tried to correct by auditing all consecutive patients discharged with “costo-chondritis”. We are also aware that retrospective audits can be flawed by recollection bias and cases might be missed by having been discharged with an alternative diagnosis. These methodological flaws could potentially be corrected by prospective studies. Also, our series was based on small-scale single centre observations in a British Overseas Territory therefore, single centre bias might apply. Scoliosis prevalence may vary by geographical areas, so larger, multi-centre cohorts might be needed to increase external validity. Mild and moderate scoliosis is often a clinical diagnosis without a “gold standard” diagnostic tool and/or a validated clinical scoring system. In the absence of such tools, the diagnosis of scoliosis might be vulnerable to observer and learning curve bias hence; structured postgraduate education and further academic work-up might be necessary.

## Conclusion

In patients presenting with sudden onset of pleuritic chest pain with reproducible chest wall tenderness, we identified a series of cases with previously undiagnosed scoliosis. We hypothesize that due to the thoracic asymmetry and the subsequent chronic changes in the functional anatomy of the spine and the rib cage; a subluxation may occur in the costo-sternal, and to a lesser extent the costo-vertebral junction. The displacement of the rib may result in pain on inspiration during certain positions/movements or even undertaking the Valsalva-manoeuvre. Recognition of scoliosis in this group of chest pain patients may help tailoring treatment with medications and non-pharmacological modalities like manipulations or exercises. If our findings are confirmed by larger multicentre, prospective observations, these patients would less likely be to subjected to unnecessary investigations, would spend less time in the overcrowded emergency department. Also, a proper education is likely to reduce the rate of unplanned returns with the same problem.

## Data Availability

See the list of references and cover letter.

## References

[1] Geyser M, Smith S (2016). Chest pain prevalence, causes, and disposition in the emergency department of a regional hospital in Pretoria. Afr J Prim Health Care Fam Med.

[2] Goodacre S, Cross E, Arnold J (2005). The health care burden of acute chest pain. Heart.

[3] Kline JA, Hernandez-Nino J, Jones AE (2007). Prospective study of the clinical features and outcomes of emergency department patients with delayed diagnosis of pulmonary embolism. Acad Emerg Med.

[4] Bĕlohlávek J, Dytrych V, Linhart A (2013). Pulmonary embolism, part I: epidemiology, risk factors and risk stratification, pathophysiology, clinical presentation, diagnosis and nonthrombotic pulmonary embolism. Exp Clin Cardiol.

[5] Crane S, Jaconelli T, Eragat M (2018). Retrospective validation of the pulmonary embolism rule-out criteria rule in 'PE unlikely' patients with suspected pulmonary embolism. Eur J Emerg Med.

[6] Robert-Ebadi H, Mostaguir K, Hovens MM, Kare M, Verschuren F, Girard P (2017). Assessing clinical probability of pulmonary embolism: prospective validation of the simplified Geneva score. J Thromb Haemost.

[7] Douma RA, Gibson NS, Gerdes VE, Büller HR, Wells PS, Perrier A (2009). Validity and clinical utility of the simplified Wells rule for assessing clinical probability for the exclusion of pulmonary embolism. Thromb Haemost.

[8] Hugli O, Righini M, Le Gal G, Roy PM, Sanchez O, Verschuren F (2011). The pulmonary embolism rule-out criteria (PERC) rule does not safely exclude pulmonary embolism. J Thromb Haemost.

[9] Freund Y, Rousseau A, Guyot-Rousseau F (2015). PERC rule to exclude the diagnosis of pulmonary embolism in emergency low-risk patients: study protocol for the PROPER randomized controlled study. Trials.

[10] Bruyninckx R, Van den Bruel A, Hannes K, Buntinx F, Aertgeerts B (2009). GPs' reasons for referral of patients with chest pain: a qualitative study. BMC Fam Pract.

[11] Epstein SK, Huckins DS, Liu SW, Pallin DJ, Sullivan AF, Lipton RI (2012). Emergency department crowding and risk of preventable medical errors. Intern Emerg Med.

[12] Menzies SM (2005). Chest wall tenderness does not exclude pulmonary embolism. Thorax.

[13] Azeredo TR, Guedes HM, Rebelo de Almeida RA, Chianca TC, Martins JC (2015). Efficacy of the Manchester triage system: a systematic review. Int Emerg Nurs.

[14] Hess EP, Agarwal D, Chandra S, Murad MH, Erwin PJ, Hollander JE (2010). Diagnostic accuracy of the TIMI risk score in patients with chest pain in the emergency department: a meta-analysis. CMAJ..

[15] Janicki JA, Alman B (2007). Scoliosis: review of diagnosis and treatment. Paediatr Child Health.

[16] The Joanna Biggs Institute tools for use in JBI Systematic Reviews. Checklist for Case Series. The Joanna Biggs Institute. 2019. [Online] Available at: https://jbi.global/sites/default/files/2019-05/JBI_Critical_Appraisal-Checklist_for_Case_Series2017_0.pdf

[17] Konieczny MR, Senyurt H, Krauspe R (2013). Epidemiology of adolescent idiopathic scoliosis. J Children's Orthoped.

[18] Malhotra R, Murali-Ganesh R, Dunkley C (2012). Acute scoliosis in a 3-year-old boy. BMJ Case Rep.

[19] Atwal R, Stewart C (2018). Acute scoliosis as an unusual presentation of pneumonia: a case report. Medicine (Baltimore).

[20] Zhu F, Chu WC, Sun G, Zhu ZZ, Wang WJ, Cheng JCY (2011). Rib length asymmetry in thoracic adolescent idiopathic scoliosis: is it primary or secondary?. Eur Spine J.

[21] Udermann BE, Cavanaugh DG, Gibson MH, Doberstein ST, Mayer JM, Murray SR (2005). Slipping rib syndrome in a collegiate swimmer: a case report. J Athl Train.

[22] Khan NAJ, Waseem S, Ullah S, et al. Slipping rib syndrome in a female adult with longstanding intractable upper abdominal pain. Case Rep Med. 2018[Online] Available at. 10.1155/2018/7484560. 10.1155/2018/7484560 PMC6051074 30057619

[23] Yao X, Blount TJ, Suzuki N, Brown LK, van der Walt CJ, Baldini T (2012). A biomechanical study on the effects of rib head release on thoracic spinal motion. Eur Spine J.

[24] Foley CM, Sugimoto D, Mooney DP, Meehan WP, Stracciolini A (2019). Diagnosis and treatment of slipping rib syndrome. Clin J Sport Med.

[25] Jenab Y, Haghani S, Jalali A (2015). Unscheduled return visits and leaving the chest pain unit against medical advice. Iran Red Crescent Med J.

